# TiO_2_ Fibers Supported β-FeOOH Nanostructures as Efficient Visible Light Photocatalyst and Room Temperature Sensor

**DOI:** 10.1038/srep10601

**Published:** 2015-06-01

**Authors:** Ting Zhu, Wei Li Ong, Liangliang Zhu, Ghim Wei Ho

**Affiliations:** 1 Department of Electrical and Computer Engineering, National University of Singapore, 4 Engineering Drive 3, 117583, Singapore; 2 Engineering Science Programme, National University of Singapore, 9 Engineering Drive 1, 117575, Singapore; 3 Institute of Materials Research and Engineering, A*STAR (Agency for Science, Technology and Research), 3 Research Link, 117602, Singapore

## Abstract

Hierarchical heterostructures of beta-iron oxyhydroxide (β-FeOOH) nanostructures on electrospun TiO_2_ nanofibers were synthesized by a facile hydrothermal method. This synthesis method proves to be versatile to tailoring of β-FeOOH structural design that cuts across zero-dimensional particles (TF-P), one-dimensional needles (TF-N) to two-dimensional flakes (TF-F). In addition, synthesizing such oxyhyroxide nanostructures presents the advantage of exhibiting similar functional performances to its oxides counterpart however, without the need to undergo any annealing step which leads to undesirable structural collapse or sintering. The as-prepared hierarchical heterostructures possess high surface area for dye adsorptivity, efficient charge separation and visible photocatalytic activity. Also, for the first time, hydrogen gas sensing has been demonstrated on β-FeOOH nanostructures at room temperature. The reported hierarchical heterostructures of β-FeOOH on TiO_2_ nanofibers afford multiple functions of photocatalysis and sensing which are highly promising for environment monitoring and clean up applications.

Among various semiconductors, titanium (IV) dioxide (TiO_2_) has been known as an excellent material for photocatalytic purposes due to its unique characteristics in band position, surface structure, as well as its chemical stability and non-toxicity^1^,^2^,^3^,^4^. There are several ways to synthesize TiO_2_ and one of it is by electrospinning. Electrospinning is a simple and versatile technique that is capable of producing nanofibers with diameters ranging from 50 to 500 nm^5^. This preparation method has the advantages of easy deposition and versatility in the synthesis of polymers, composites and ceramics. Due to their nanosize, nanofibers possess a range of attractive properties such as high surface area to volume ratio, flexibility of structures and mechanical integrity^6^. The formation of nanofibers with TiO_2_ allows a combination of their elevated surface area and the intrinsic properties of TiO_2_, thereby opening an enormous potential in this material for applications in environmental remediation and protection, photocatalysis, dye-sensitized solar cells, gas sensors, and batteries^7^,^8^,^9^,^10^,^11^.

However, TiO_2_ is only able to utilize the photons in the UV region (λ < 380 nm) due to its large band gap (E_g_ = 3.2 eV), severely limiting its practical applications in sun light and indoor environment^12^,^13^,^14^. A possible strategy to overcome this drawback is to couple TiO_2_ with narrow bandgap semiconductors capable of harvesting the photons in the visible range^15^,^16^,^17^. Fe_2_O_3_ is considered to be a suitable semiconductor to be coupled with TiO_2_ due to its high photocatalytic activity and approximate band gap energy as compared with TiO_2_^18^,^19^,^20^,^21^. Fe_2_O_3_ can be prepared by the forced hydrolysis of Fe(III) solutions where iron oxyhydroxides (α-FeOOH and β-FeOOH) are intermediate products, and transform to Fe_2_O_3_ through post treatment^22^,^23^,^24^,^25^.

The intermediate product β-FeOOH (*E*_g_ = 2.12 eV)^26^ is actually capable of showing photocatalytic activity in the visible range. β-FeOOH has a channel structure parallel to the c-axis and this tunnel structure makes β-FeOOH an especially interesting material which is promising as a photo-Fenton catalyst in the heterogeneous system^27^,^28^,^29^. β-FeOOH is an ideal material to couple with the TiO_2_ nanofibers for photocatalytic activity in the visible range because it exhibits the same performance as Fe_2_O_3_ but has a simpler synthesis process where unlike Fe_2_O_3_, it does not have to undergo the annealing process which may lead to the collapse of hierarchical structures. Moreover, a variety of morphologies can be easily obtained without the use of surfactant. However, there are very few reports of β-FeOOH-TiO_2_ hierarchical nanostructures. Hierarchical and branched nanostructures possess the combined advantages of rapid charge transfer pathway for carrier collection, large surface area for increased reaction sites, and excellent light trapping^30^,^31^,^32^ which are all favorable characteristics for photocatalytic reactions. The large surface area of the structure also makes it highly suitable for gas sensing applications.

In this work, we have synthesized β-FeOOH on TiO_2_ nanofibers and 3 different types of nanostructures were easily obtained by adjusting the concentration of the FeCl_3_ aqueous solution without using any surfactants. TiO_2_@FeOOH in the forms of flakes (TF-F), particles (TF-P) and needles (TF-N) were prepared with [FeCl_3_] = 0.02, 0.05 and 0.1 M, respectively. The as-obtained products are unique in structure and porous in texture, and are evaluated for their methyl orange (MO) dye degradation and hydrogen (H_2_) gas sensing performances. The samples displayed enhanced photodegradation capabilities and gas sensing at room temperature. The TiO_2_@FeOOH composites also proved to be active in visible light and were able to degrade MO under visible light illumination.

## Results and Discussion

**Figure 1:**
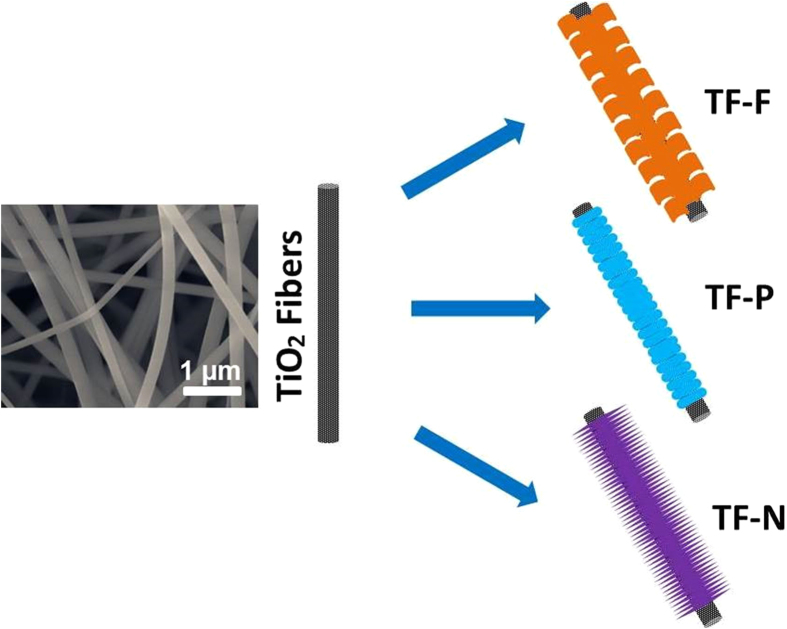
FESEM image of TiO_2_ fiber and the formation scheme of the Samples TF-F, TF-P, and TF-N.

The synthesis of FeOOH architecture onto the TiO_2_ nanofibers is illustrated in Fig. 1. The electrospun TiO_2_ nanofibers with an average diameter of 200 nm served as hard template for the growth of different FeOOH hierarchical structures. By controlling the concentrations of FeCl_3_ solution, FeOOH with different architectures can be fabricated through a facile hydrothermal process without introducing any surfactants. TiO_2_@FeOOH in the forms of flakes, particles and needles were obtained with [FeCl_3_] = 0.02, 0.05 and 0.1 M, respectively. The as-obtained products have shown to be unique in structure and porous in texture, and are evaluated for their dye degradation and H_2_ gas sensing performances.

**Figure 2:**
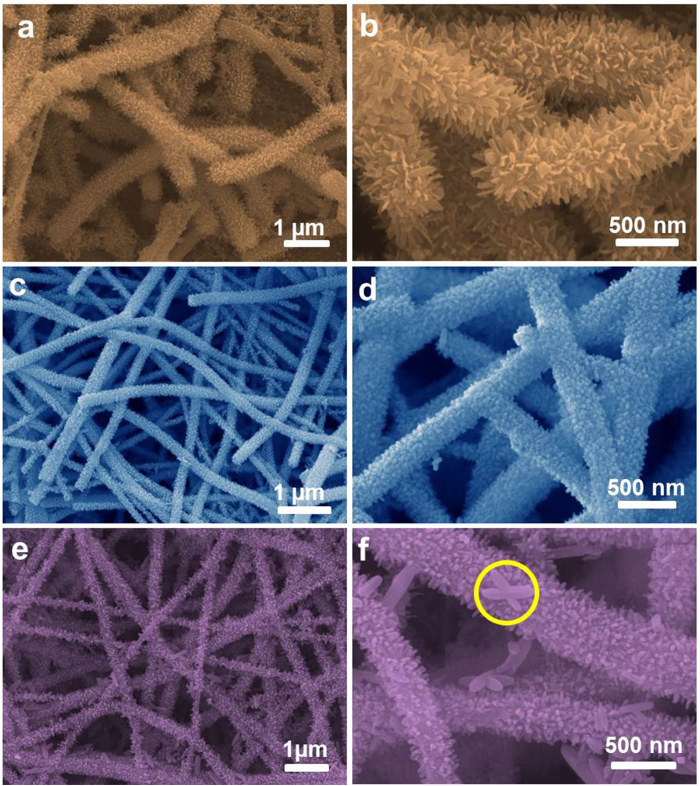
FESEM images of Samples TF-F (**a** and **b**), TF-P (**c** and **d**), and TF-N (**e** and **f**).

**Figure 3:**
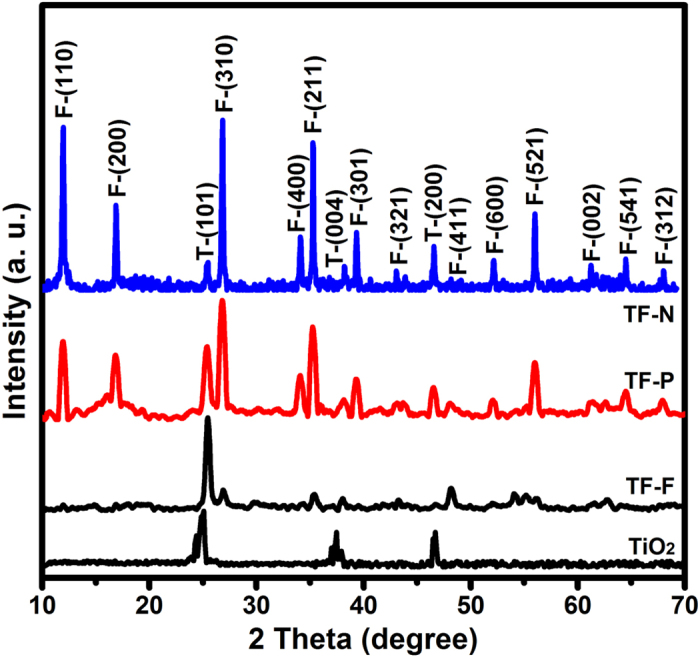
XRD patterns of the Samples TF-F, TF-P, and TF-N.

The pristine TiO_2_ nanofibers are shown in the SEM image in Fig. 1, where a diameter of ca. 200 nm and smooth surface can be clearly observed. After the growth of FeOOH architectures, the morphologies of TiO_2_@FeOOH are as shown in Fig. 2. With the lowest concentration of FeCl_3_ in this work, ultrafine nanoflakes were formed on the TiO_2_ fibers, thickening the fiber diameters up to 500 nm (Fig. 2a,b). When [FeCl_3_] was increased to 0.05 M, the flake-like structure disappeared and changed to nanoparticles. The diameter of the fibers was increased from 200 nm to 300 nm, indicating a thickness of 50 nm of the particle-like FeOOH layer (Fig. 2c,d). The further increase of FeCl_3_ to 0.1 M leads the particles to grow longer into needles, which are revealed by SEM results in Fig. 2e,f. The diameters of the cables are measured to reach 500 nm again, which marked the length of the needles at ~60 nm. However, it is interesting to find some cross-shaped particles appearing amongst the sample (marked in yellow circle), which may be due to over-crystallization caused by the higher concentration of Fe^3+^. The crystallographic phases of all the as-obtained TiO_2_@FeOOH samples together with the pure TiO_2_ (as a comparison) were examined by XRD (Fig. 3). All the indexed peaks marked with F can be assigned to β-FeOOH (JCPDS 75-1549)^33^, while the peaks marked with T can be attributed to anatase TiO_2_ (JCPDS 21-1272)^34^. No peaks from other impurities can be detected which shows the successful deposition of FeOOH on the TiO_2_ fibers.

**Figure 4:**
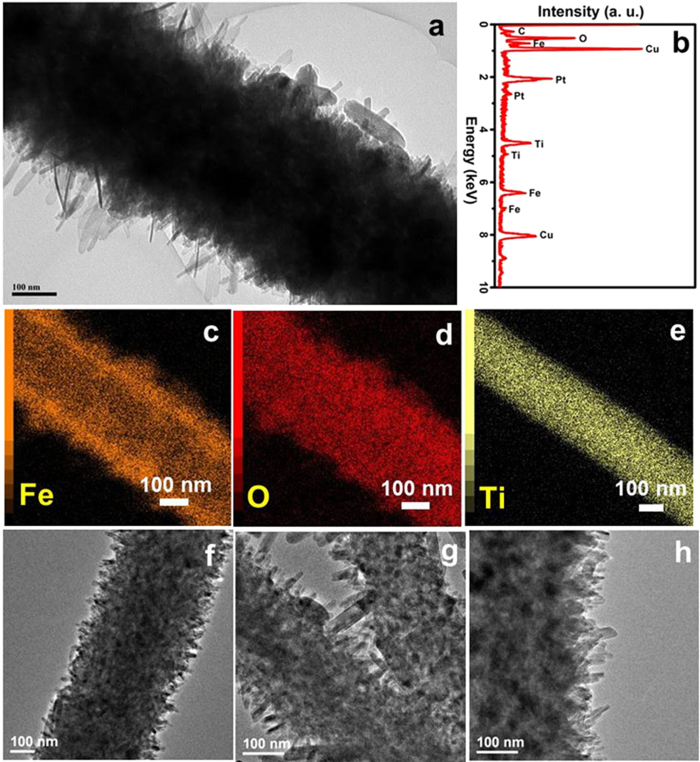
TEM (**a**, **f**, **g** and **h**), EDX (**b**) and elemental mappings (**c**, **d** and **e**) of the Sample TF-F (**a**-**e**), TF-P (**f**) and TF-N (**g** and **h**).

The detailed morphologies and compositions of the as-fabricated TiO_2_@FeOOH nanocables are further examined by TEM and elemental mapping. Figure 4a shows a TEM image of the Sample TF-F, where ultrafine nanoflakes growing from the fiber can be observed. The diameter of the nanocable is estimated to be around 500 nm, which is consistent with the previous SEM findings. The chemical composition of the TF-F is revealed by the EDX result shown in Fig. 4b, where the presence of Fe, Ti, O and C is confirmed (the peaks of Cu and Pt are attributed to the Cu substrate and Pt sputtering for SEM characterization respectively). In order to further analyze the typical core-shell structure of the TF-F nanocables, elemental mapping was also performed. The mappings of Fe, O and Ti are presented in Fig. 4c to e, clearly demonstrating the core-shell structure of the cables, where TiO_2_ fiber is encapsulated by the FeOOH architecture. Furthermore, no Ti is detected in the shell structure which shows that Ti has been confined within the cable cores, ensuring the mechanical stability of the entire structure. As a comparison, the TEM results of Sample TF-P and TF-N are also presented in Fig. 4 to show the differences of all the three samples. The TF-P sample is shown as Fig. 4f, in which tiny particles can be observed along the cable structure, compared to the much longer needle-like structure for sample TF-N (Fig. 4g,f).

**Figure 5:**
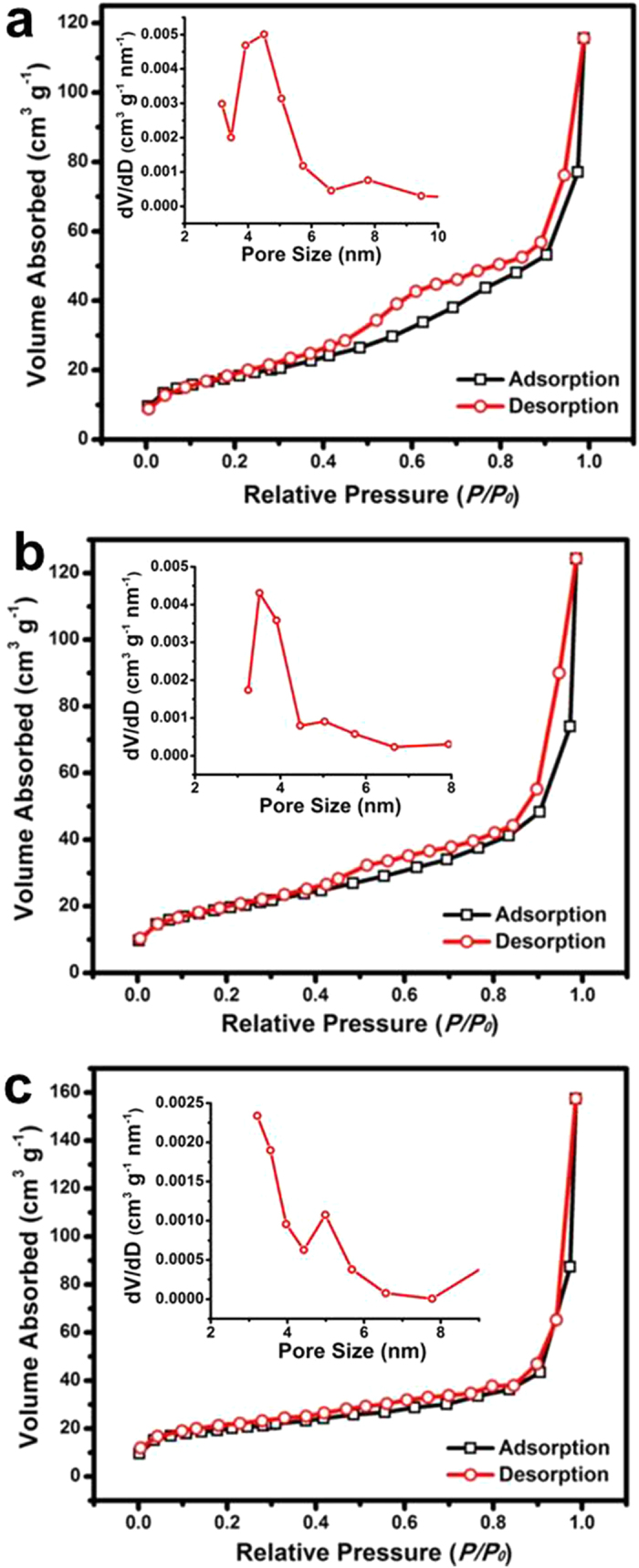
BET results of the Sample TF-F (**a**), TF-P (**b**), and TF-N (**c**). The insets show the corresponding pore size distributions obtained from desorption isotherms.

The BET measurements were performed at 77 K to investigate the textural characteristics of all the three samples. N_2_ adsorption-desorption isotherms of all the samples are shown in Fig. 5 with insets illustrating their corresponding pore size distributions obtained from respective desorption branches. These isotherms can be categorized as type IV with small hysteresis loops observed at a relative pressure of 0.4-0.9 for all the three samples. The BET specific surface areas are calculated to be 67, 21 and 64 m^2^ g^−1^ for samples TF-F, TF-P and TF-N, respectively, showing the porous texture of all the samples. It can be concluded from the pore size distributions that TF-P and TF-N have pores with diameters of ~3.4 and 3.2 nm, respectively, while TF-F possesses a larger pore size at ~4.4 nm. In virtue of the porous texture and high surface area, the as-obtained TiO_2_@FeOOH core-shell nanocables would provide more active sites for photocatalytic reactions and gas absorption compared to common materials.

**Figure 6:**
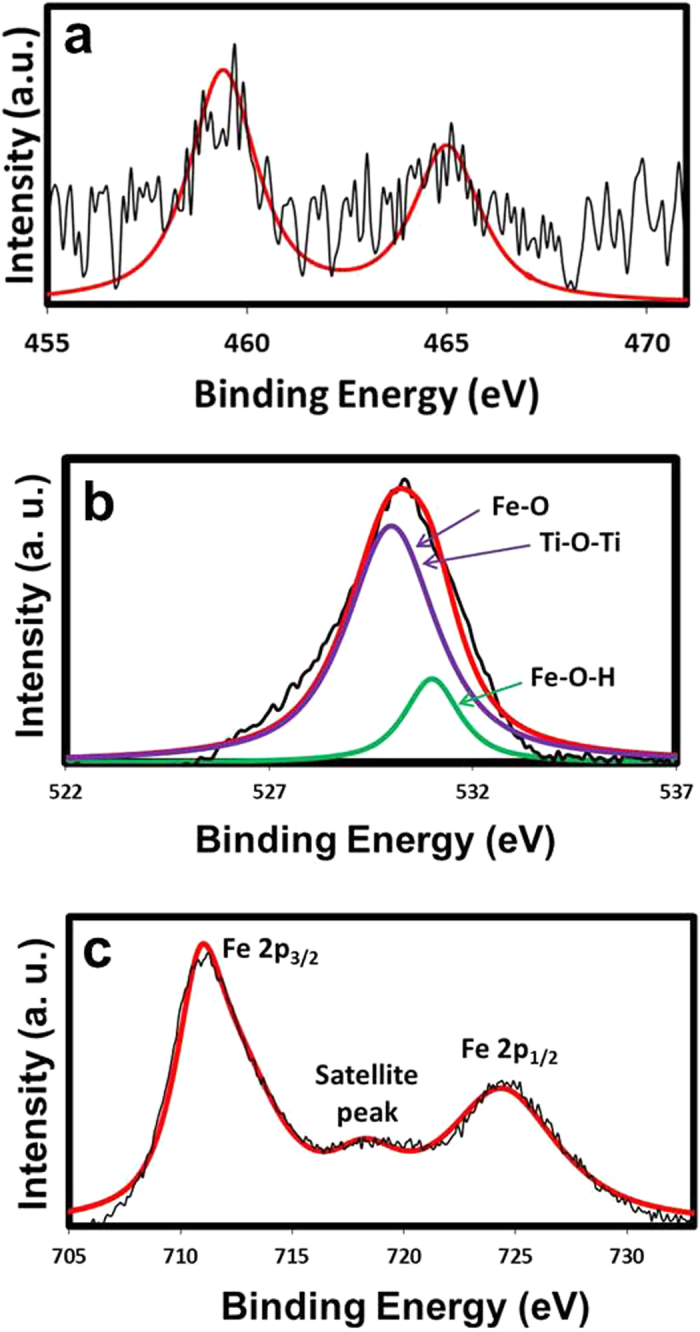
XPS results of the typical TF-F sample.

The elemental composition of the TiO_2_@FeOOH nanocables is shown by the XPS spectra in Fig. 6. The peaks of Ti 2p_3/2_ at 459.4 eV and Ti 2p_1/2_ at 465.0 eV indicate the presence of Ti^4+^ and that Ti is present in the form of TiO_2_^35^. The O1s peak can be deconvoluted into 2 peaks located at 530 and 531 eV. The peak at 531 eV corresponds to the Fe-O-H bond^36^ while the peak at 530 eV can be attributed to both the Fe-O^36^ and Ti-O-Ti bonds^37^. The presence of Fe-O and Fe-O-H bonds suggest the formation of FeOOH while the Ti-O-Ti bond indicates the presence of TiO_2_. The formation of FeOOH is also proven by the Fe 2p_3/2_ and Fe 2p_1/2_ peaks at 711 and 724.4 eV respectively which corresponds to Fe^3+^ 38. The Fe 2p_3/2_ satellite peak at 719 eV is a characteristic peak of Fe^3+^, similar to other iron oxide samples of only Fe^3+^ states^36^. The XPS results of the typical TF-F sample after UV-vis photocatalysis is also provided as Figure S1 (see in the Supplementary Information), where no peak shifts can be observed compared to that before photocatalytic reaction.

**Figure 7:**
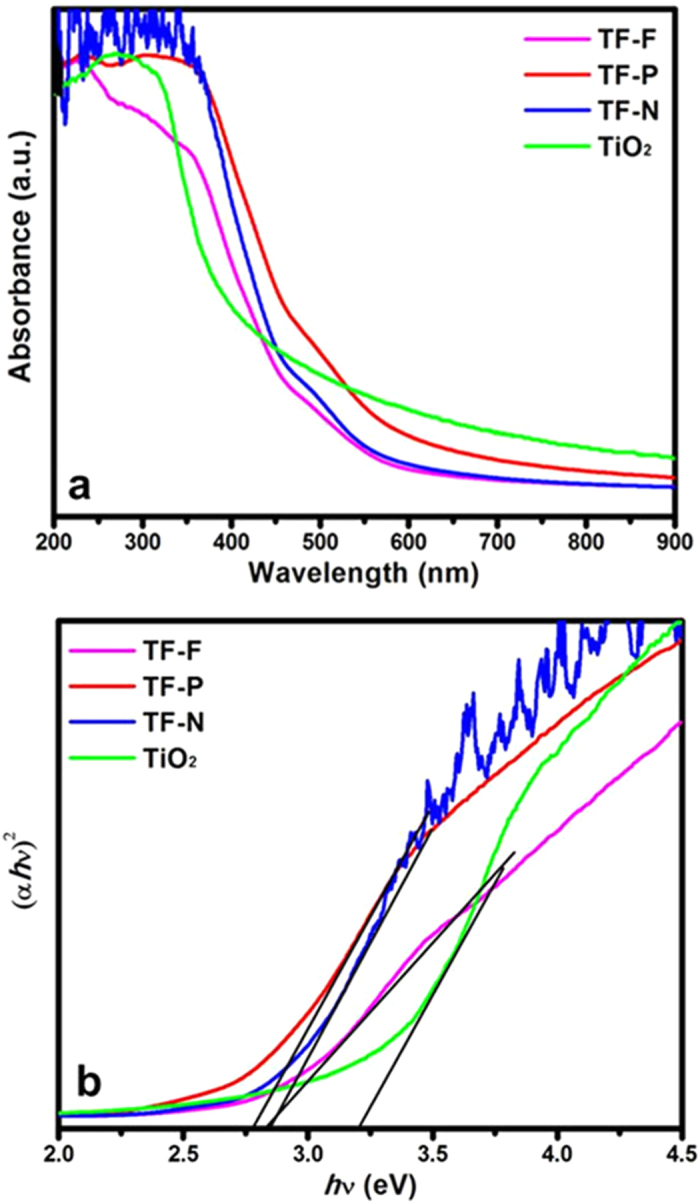
(**a**) UV-vis absorbance spectra and Tauc plot of the TF samples.

The UV-vis absorbance spectra of the TiO_2_@FeOOH nanocables are shown in Fig. 7a. The TiO_2_ nanofibers display photoabsorption in the UV region with an absorption edge at about 380 nm, but this absorption edge shifted to higher wavelengths with the addition of FeOOH. From the extrapolation of the straight line region of the Tauc plots in Fig. 7b, the bandgap is estimated to be 3.2 eV for TiO_2_ nanofibers. The bandgaps of the TiO_2_@FeOOH nanocables are narrower at 2.75, 2.8 and 2.81 eV for the TF-P, TF-F and TF-N respectively. The narrowing of bandgap is due to the coupling of TiO_2_ with β-FeOOH. The formation of Fe-O-Ti bonds will overlap the conduction band of TiO_2_ and *d*-orbital of Fe^3+^ 39, allowing the composite to harness visible light for photocatalytic reactions.

The most commonly used photocatalyst for dye degradation is TiO_2_ due to its low cost, high catalytic activity and long-term stability^40^. Han *et al.* reported the synthesis of TiO_2_ nanosheets with (001) facets for photocatalytic MO degradation under UV light, and the photocatalytic results have shown the superiority of their TiO_2_ nanosheets over the commercial P25^41^. A later work by Sun *et al.* has developed a TiO_2_-Graphene composite for improved UV-light photocatalytic activity. In their experiments, the dye degradation property of P25, TiO_2_ nanotubes, TiO_2_ nanosheets are also compared to show the enhanced performance of the as-prepared TiO_2_-Graphene composite^42^. In addition, the morphological investigation is also performed to study the relationship between the photocatalytic property and material structures. A recent work by Fu *et al.* has demonstrated the synthesis of TiO_2_ with different shapes with different performances in photocatalytic MO degradation^43^. Unfortunately, TiO_2_ is a wide-bandgap semiconductor, which is active under UV light only, limiting its harvesting capability for solar energy. Also, hydrofluoric acid (HF, extremely corrosive) is employed in some of the method for TiO_2_ synthesis, making those methods not so practical^41^,^43^. However, TiO_2_@FeOOH for visible-light photocatalysis is rarely reported. In our work, TiO_2_ nanofibers are obtained by an electrospin method, involving no toxic or corrosive chemicals. Furthermore, β-FeOOH materials with different nanostructures are grown onto the TiO_2_ nanofibers to form the TiO_2_-FeOOH hybrids, which are proven to be sensitive to the visible light irradiation. Hence, the as-fabricated TiO_2_ supported β-FeOOH is able to degrade the MO under the whole spectrum of sun light, which could be a more efficient photocatalyst.

**Figure 8:**
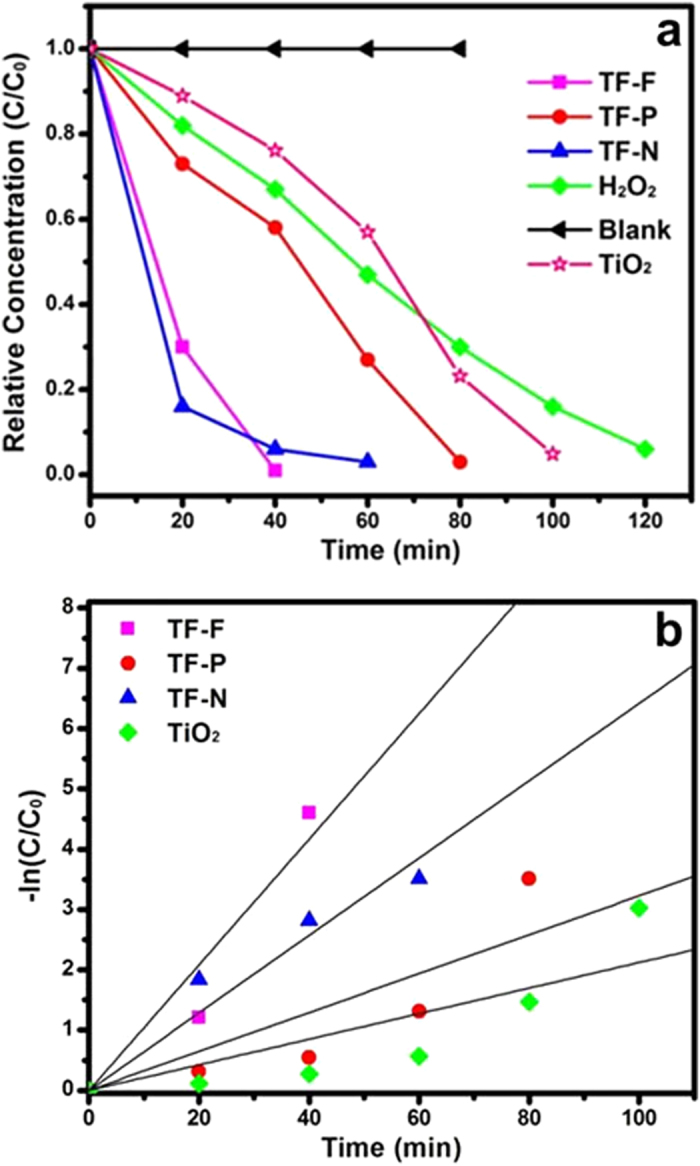
(**a**) Degradation kinetics and (**b**) pseudo-first order kinetics of time evolution MO photodegradation study in the presence of TF samples under UV-visible illumination.

The photocatalytic degradation of MO was used to demonstrate the enhanced photocatalytic activity of the TiO_2_@FeOOH nanocables. The degradation kinetics of MO was measured via the changes in their concentration, which was calculated from the absorbance peaks. Figure 8a shows the time profiles of the decrease in MO concentrations in the presence of TiO_2_@FeOOH nanocables under UV-Vis illumination. A blank experiment was carried out to show that photodegradation is not apparent in the absence of photocatalyst and H_2_O_2_. When H_2_O_2_ was added, photodegradation of MO was relatively slow and the reaction was completed only after 120 min of irradiation. With the addition of TiO_2_ nanofibers and H_2_O_2_, the MO could be fully degraded within 100 min. Comparatively, the TiO_2_@FeOOH nanocables showed enhanced photocatalytic activity under UV-Vis irradiation and were able to fully degrade the MO dye after 40-80 min, albeit at varying rates. The photodegradation activity was further analyzed by studying the pseudo-first order kinetics of the various photocatalysts as shown in Fig. 8b. This quantitative analysis is derived using the pseudo-first order model^44^ as follows:
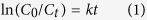
where *C*_0_ and *C*_*t*_ are the concentrations of MO at time 0 and *t* respectively, and *k* is the pseudo-first order rate constant.

The pseudo-first order rate constants, *k*, of the plain TiO_2_ nanofibers and TiO_2_@FeOOH nanocables are summarised in Table 1. The constant *k* of TF-F is the highest at 0.1041 min^−1^ while that of TF is the lowest at 0.0212 min^−1^. The enhancement in photodegradation exhibited by the TiO_2_@FeOOH nanocables can be attributed to the heterogeneous photo-Fenton-like process, where large amounts of free hydroxyl radicals are rapidly generated from the reaction between FeOOH and H_2_O_2_ under UV-Vis irradiation^45^. Peroxide complex species are initially formed at Fe^3+^ active sites on the surface of the catalyst. The iron complex then undergoes a cleavage by UV-Vis irradiation to form Fe^2+^ complex. The unstable Fe^2+^ complex is rapidly oxidized by H_2_O_2_ to form hydroxyl radicals which could degrade MO^38^. Furthermore, under UV-Vis irradiation, electron-hole pairs are generated on the TiO_2_@FeOOH surface. The excited electrons will transfer from the conduction band of FeOOH to that of TiO_2_. This in turn results in reduced recombination, and the long-lived charge separated states promote generation of photoreactive oxidative species, *i.e.*^·^O_2_^−^ and OH^·^^46^,^47^, which are responsible for degrading MO. OH^·^ radicals are also formed from the reduction of H_2_O_2_ by the trapped electrons and via self-decomposition by UV-Vis illumination, as shown below^48^,^49^:
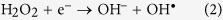

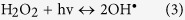


The degradation results clearly show that TF-F has the best photocatalytic performance amongst the TiO_2_@FeOOH nanocables. This is most likely attributed to the high surface area of TF-F (67 m^2^/g) as compared to TF-N (64 m^2^/g) and TF-P (21 m^2^/g). With a larger surface area, TF-F has more sites for the photocatalytic degradation of MO compared to the other composites. Moreover, the FeOOH flakes have a 2D structure while FeOOH needles are 1D and FeOOH particles are 0D. 2D structures generally have better charge transport properties than 1D and 0D structures^50^, thus the chances of recombination and trapping during the transport of photoexcited electrons from the conduction band of FeOOH to that of TiO_2_ is also reduced. As a result, more photoreactive oxidative species are produced, leading to enhanced MO photodegradation rate. To investigate the stability of the TF samples, the TF-N sample was re-collected and characterized by XRD. The peak-match results have shown that there is no phase change after irradiation for up to 3 h, indicating the stability of the materials (Figure S2).

The degradation kinetics of all the three TF samples without the addition of H_2_O_2_ are also carried out and the results are shown in Figure S3, from which degradation processes are observed to be much slower and less efficient compared to the cases with H_2_O_2_ present.

**Figure 9:**
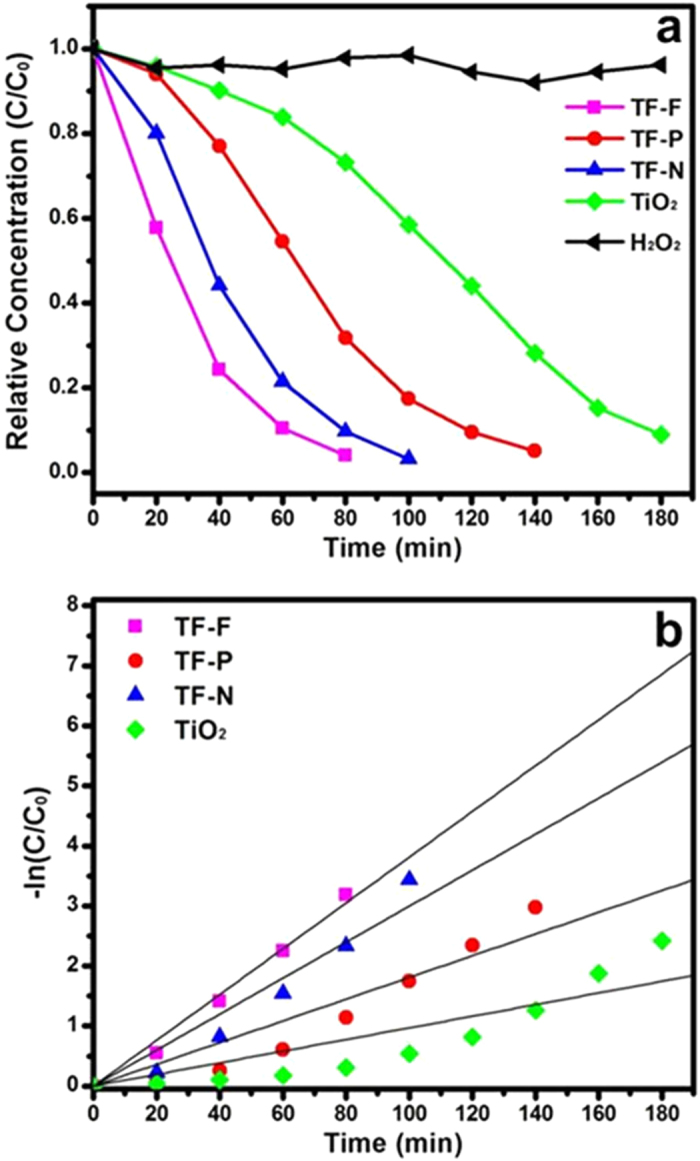
(**a**) Degradation kinetics and (**b**) pseudo-first order kinetics of time evolution MO photodegradation study in the presence of TF samples under visible illumination.

Besides photodegradation of MO under UV-Vis illumination, the TiO_2_@FeOOH nanocables are also capable of degrading MO under visible illumination (Fig. 9a). Contrary to that of UV-Vis illumination, no significant degradation of MO was observed when only H_2_O_2_ was added to MO. This is because self-decomposition of H_2_O_2_ does not occur under visible illumination, and hence, no OH^·^ radicals were formed to degrade MO. When TiO_2_ nanofibers were added, the MO was degraded after 180 min of visible illumination. The photocatalytic activity in visible illumination can probably be attributed to photoreaction of the peroxide complex that is formed on the TiO_2_ surface. During this process, an electron is transferred from the photoexcited complex to the conduction band of TiO_2_, and then transferred to H_2_O_2_, leading to the generation of an OH^−^ ion and an OH^·^ radical which can degrade MO^51^. The degradation was even faster when TiO_2_@FeOOH nanocables were used, where TF-F degraded the MO in 80 min with a constant *k* of 0.0381 min^−1^ as shown in Fig. 9b and Table 2. The increase in degradation time of 40 to 60 min for all the samples can be attributed to the reduced amount of electron-hole pairs generated in visible light since electron-holes pairs could be generated from FeOOH and less effectively from TiO_2_. In order to study the adsorption property of the as-prepared TF samples, the adsorption kinetics for all the three TF samples without light irradiation are presented in Figure S4. A physical adsorption may have occurred for all the three samples in the first 60 minutes possibly due to the electrostatic force and high surface areas. Thereafter, some adsorbed MO molecules desorbed and re-enter into the solution with effect of the magnetic stirring (the C/C_0_ values recovered to 0.27, 0.56 and 0.42 for TF-F, P and N). This has shown a good adsorption property of our materials, which is advantageous to photocatalytic degradation. It also should be noted that the C/C_0_ values without light irradiation are still much higher than those in the presence of visible or UV light, which clearly show the photocatalytic activities of the as-prepared TF samples.

In the gas sensing, hematite (Fe_2_O_3_) is extensively used by the research community because of the thermodynamic stability and abundant in earth^52^. Many efforts have been focused on the synthesis of nanostructured Fe_2_O_3_ with different shapes and morphologies. Wang *et al.* have reported Fe_2_O_3_ nanowires for gas sensing application and their obtained results have presented better performance of the Fe_2_O_3_ nanowires over the commercial Fe_2_O_3_ powder^53^. In another work by Hao *et al.*, different hierarchical Fe_2_O_3_ architectures were prepared using various synthetic conditions. The gas sensing property of different Fe_2_O_3_ architectures were evaluated and compared^54^. In recent years, hematite based nanocomposites were developed to improve the gas sensing performance. For example, Ag-α-Fe_2_O_3_ composite material was reported by Liu *et al.* through a facile solution-based method. The Ag loaded hematite has shown the best performance when compared to the unloaded Fe_2_O_3_ and Fe_2_O_3_ nanocubes^55^. However, rare reports have been seen for the gas sensing using FeOOH as straightforward sensor materials. Herein we have grown the β-FeOOH onto the TiO_2_ nanofibers to create the TF hybrid materials with high specific surface areas. The as-derived TF materials have been used as gas sensor electrode directly without converting them to Fe_2_O_3_ by heat treatment, which may free the materials from morphological changes or structural collapse. In addition, the TF hybrid materials-based gas sensors are capable of functioning at room temperature and shows better sensing performance than some of the Fe_2_O_3_ gas sensors reported in literature^56^,^57^. The gas sensors reported by Flak *et al.*^56^ and Long *et al.*^57^ are operated at 300 °C and they show sensitivities of less than 8 in 500 ppm of H_2_.

**Figure 10:**
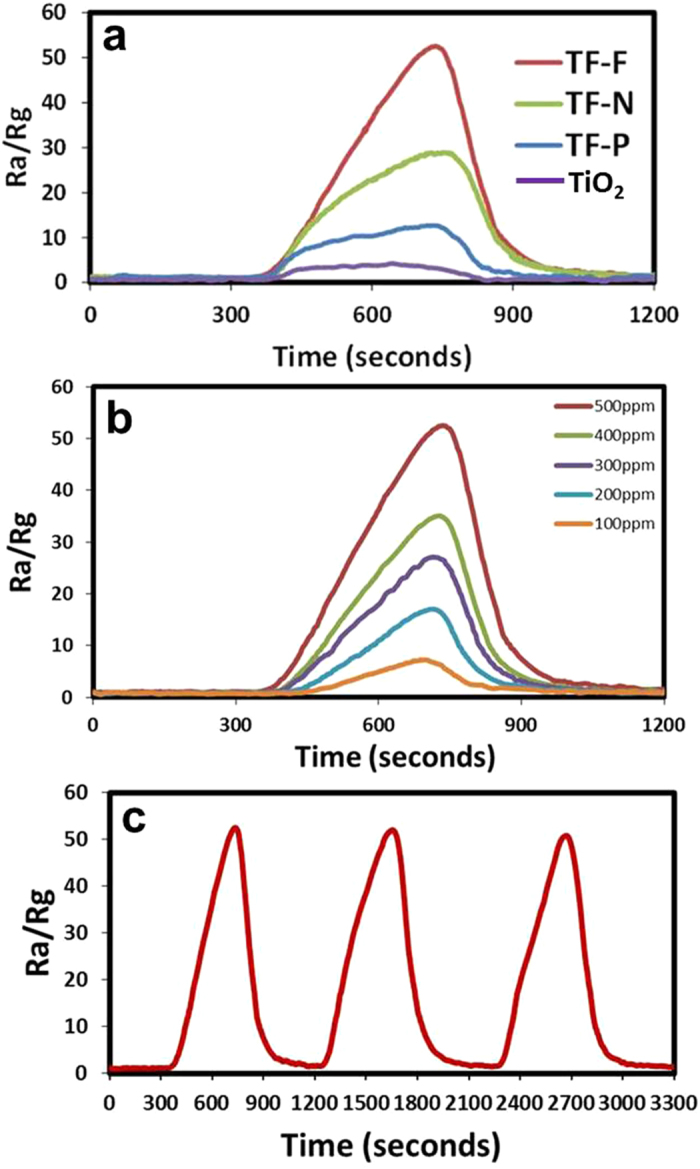
(a) Sensitivity of the prepared TF samples at 500 ppm of hydrogen . (**b**) Sensitivity of the prepared TF-F sample as the gas ambient is switched from air to various concentrations (100–500 ppm) of hydrogen at room temperature. (**c**) Dynamic gas response (*R*_*a*_*/R*_*g*_ ) of the prepared TF-F sample at 500 ppm.

The H_2_ sensing properties of TiO_2_@FeOOH nanocables at room temperature are also investigated. The samples are placed in a homemade gas sensing setup where the ambient in the sensing chamber is switched periodically between CDA and H_2_ gas of various concentrations (100 to 500 ppm). The resistance of the samples are observed to decrease in H_2_ ambient and increase in CDA ambient. The sensitivity of the sensor is calculated by
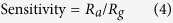
where *R*_a_ is the resistance of the sample in CDA and *R*_g_ is the resistance of the sample in H_2_ gas. The TiO_2_@FeOOH nanocables were tested and their sensing responses to H_2_ gas are plotted in Fig. 10a. It is evident that the composites are all capable of sensing H_2_ gas at room temperature, with TF-F exhibiting the highest sensitivity of 52.5 in 500 ppm of H_2_ gas. TF-N has a sensitivity of 28.9 while TF-P showed a lower sensitivity of 12.7. TiO_2_ nanofibers have the lowest sensitivity of 4.

The H_2_ gas sensing mechanism of the TiO_2_@FeOOH nanocables can be explained by the adsorption and desorption of O_2_ molecules at the surface of the TiO_2_@FeOOH nanocables. When the TiO_2_@FeOOH nanocables are exposed to CDA, the O_2_ molecules present in the air will adsorb onto the surface of the TiO_2_@FeOOH nanocables:
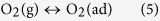


The physisorbed O_2_ molecules will trap electrons from the conduction band of the TiO_2_@FeOOH nanocables to form chemisorbed O_2_ species of O^−^, O^2−^ and O_2_^−^, with O_2_^−^ being favorably chemisorbed at room temperature^58^,^59^:
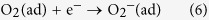


The trapping of electrons results in the formation of an electron depleted region at the surface of the TiO_2_@FeOOH nanocables. This depletion of electrons from the conduction band causes the resistance of the TiO_2_@FeOOH nanocables to increase. It also causes a surface potential barrier to form in the space charge region, inhibiting the electron flow across the grain boundaries, thus increasing the resistance of the interlinked network of TiO_2_@FeOOH nanocables. However, when the TiO_2_@FeOOH nanocables are exposed to H_2_ gas, the H_2_ molecules will react with the chemisorbed oxygen ions to form water molecules and desorb from the surface of the nanostructures, releasing the trapped electrons back to the conduction band of TiO_2_@FeOOH nanocables in the process.
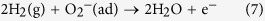


The increase in the number of electrons in the conduction band leads to a reduction in the width of the depletion region and the height of the surface potential barrier, causing the resistance of the sensor to decrease^60^. When the environment in the sensing chamber is reverted back to CDA, the O_2_ molecules adsorbed onto the surface of the TiO_2_@FeOOH nanocables once again, creating a depletion region and causing the resistance of the sample to increase. The sensitivity of the material depends largely on the specific surface area and pore size. With the same surface area, larger pores can ensure a higher sensitivity due to enhanced diffusion of the gas^61^. The TF-F exhibited the highest sensitivity to H_2_ gas because it has the highest surface area and largest pore size amongst the three TiO_2_@FeOOH nanocables. This result in faster diffusion and a larger surface area being available for reaction with the gas molecules and thus, a greater change in the resistance of the sample will be obtained. The TF-F sample sensor exhibited a sensitivity of 52.5 at 500 ppm of H_2_ gas, and is able to sense hydrogen gas down to 100 ppm with a sensitivity of 7.3 (Fig. 10b). The sample displayed discrete sensitivity values for each H_2_ gas concentration, demonstrating the ability to differentiate and quantify various H_2_ gas concentrations.

The dynamic response of the gas sensing characteristics of TF-F to H_2_ is investigated and the results are summarized in Fig. 10c. It can be observed that TF-F showed remarkably high sensitivity and reversibility. Furthermore, the reversibility and stability are demonstrated by the complete recovery of the resistance value after switching off the target gas during many cycles, suggesting the highly reversible interactions between the analytes and the sensor elements. The differences in the sensing behaviors between TF-F, TF-N and TF-P may be related to the effects of steric hindrance on the diffusion and accessibility of the target gases to the deeper region of the FeOOH layers in view of the different surface area of the nanostructures.

In summary, β-FeOOH nanostructures were grown onto electrospun TiO_2_ nanofibers by a facile hydrothermal method. Three types of FeOOH nanostructures namely 0D particles, 1D needles and 2D flakes could be obtained by varying the concentration of FeCl_3_. The TiO_2_@FeOOH nanocables have high surface areas ranging from 21-67 m^2^ g^−1^ and are capable of degrading MO under both UV-Vis and visible illumination. The samples are also able to sense various concentrations of H_2_ gas at room temperature. The TF-F sample exhibited the best performance both in the photocatalytic dye degradation and gas sensing applications.

## Methods

### Synthesis of TiO_2_ nanofibers

0.7 g of polyvinylpyrrolidone (PVP, Mw = 1 300 000) and 2 g of tetrabutyl titanate (TBT) were dissolved in 7.3 g of solvent mixture consisting of ethanol/acetic acid (4/1, v/v) by stirring for 5 h to obtain a homogeneous solution. Subsequently, electrospinning was carried out at an applied voltage of 18 kV with a flow rate of 4 mL h^−1^. The distance between needle tip and aluminum foil collector is 15  cm. The collected materials were hydrolyzed in air for 3 h. Finally, the as-spun nanofibers were calcined in air at 500 °C for 2 h in a furnace with a temperature ramp of 2 °C min^−1^ to obtain TiO_2_ nanofibers.

### Fabrication of FeOOH onto TiO_2_ nanofibers

Typically, 10 mg of TiO_2_ nanofibers were dispersed in 20 mL of FeCl_3_ aqueous solution (0.02M) by sonication for 10 min to achieve a homogeneous mixture. The mixture was then transferred into a glass bottle, sealed and heated at 90 °C for 12 h. After cooling to room temperature, the product was washed and centrifuged several times with deionized (DI) water and ethanol before drying overnight at 60 °C in an air-flow oven.

### *Materials characterization*

All the samples are characterized by field-emission scanning electron microscopy (FESEM, JEOL FEG JSM-7001F) equipped with an energy dispersive X-ray spectroscopy (EDX), transmission electron microscopy (TEM, Philips FEG CM300) equipped with elemental mapping system and X-ray diffraction (XRD, Philips X-ray diffractometer, Cu Ka). X-ray photoelectron spectroscopy (XPS), VGSCALAB 220I-XL system equipped with an Mg Kα x-ray source was employed to study the elemental compositions. The N_2_ adsorption and desorption isotherms were measured at 77 K by a Quantachrome NOVA-1200 system. The BET surface area was calculated using adsorption data in a relative pressure ranging from 0.05 to 0.3. The absorption spectra of the samples and methyl orange (MO) aqueous solutions were obtained with a UV-VIS-NIR spectrophotometer (UV-VIS, Shimadzu UV-3600). The electrical characterization for H_2_ gas sensing was carried out using a Keithley 4200-SCS semiconductor characterization system.

### *Dye degradation*

The photocatalytic activity of the as-prepared samples for the degradation of MO was evaluated by measuring the absorbance of the irradiated solution. 20 mg of TiO_2_ nanofibers or TiO_2_@FeOOH nanocables was mixed with 15 ml of MO aqueous solution (0.06 mM) and 0.5 ml of hydrogen peroxide solution (H_2_O_2_, 30-32 wt %). The suspension was magnetically stirred in the dark for 60 min to reach a complete adsorption-desorption equilibrium, before being illuminated with a 300 W Xenon arc lamp of intensity 100 mW cm^−2^. For photodegradation of MO under visible illumination, a 420 nm cutoff filter was placed in front of the light source. The concentration of MO was determined using a UV-VIS-NIR spectrophotometer and the maximal absorbance peak value (at 463 nm) was noted to plot the amount of MO degraded and thus, determine the photodegradation activity of the composite.

### *Gas sensing*

The TiO_2_ nanofibers and TiO_2_@FeOOH nanocables were mixed with polyethylene glycol (PEG, average molecular weight 400) to obtain a paste which was spread on a microscope glass slide by the “doctor blade” technique to form a film of ~10 μm in thickness. The film was dried at 100 °C for 2 h in air. H_2_ gas sensing was carried out at room temperature by applying a voltage bias of 10 V to the sample and measuring the change in resistance of the sample when the ambient in the sensing chamber was switched between clean dry air (CDA) and H_2_ gas. The sensitivity of the samples in various H_2_ concentrations (100 to 500 ppm) was measured. The various H_2_ concentrations were obtained by varying the flow rate of CDA and H_2_ gas into the sensing chamber.

## Additional Information

**How to cite this article**: Zhu, T. *et al.* TiO_2_ fibers supported ß-FeOOH nanostructures as efficient visible light photocatalyst and room temperature sensor. *Sci. Rep.* **5**, 10601; doi: 10.1038/srep10601 (2015).

## Supplementary Material

Supplementary Information

## Figures and Tables

**Table 1:** Pseudo-first order kinetics of time evolution MO photodegradation under UV-visible illumination.

**Photocatalysts**	**Kinetic constants, k (min**^**−1**^)	**Correlation coefficient, R**^**2**^
TF-F	0.1041	0.9154
TF-P	0.0323	0.7596
TF-N	0.0642	0.9309
TF	0.0212	0.7335

**Table 2:** Pseudo-first order kinetics of time evolution MO photodegradation under visible illumination.

**Photocatalysts**	**Kinetic constants, k (min**^**−1**^)	**Correlation coefficient, R**^**2**^
TF-F	0.0381	0.988
TF-P	0.0181	0.9015
TF-N	0.03	0.9372
TF	0.0097	0.7871
