# Supplementary material for: TiO2 Fibers Supported β-FeOOH Nanostructures as Efficient Visible Light Photocatalyst and Room Temperature Sensor
Source: Sci Rep. 2015 Jun 1;5:10601. doi: 10.1038/srep10601 (PMC4450542; doi:10.1038/srep10601)
Supplement: Supplementary Information [file srep10601-s1.pdf]

Supplementary Information

TiO_2 Fibers Supported β-FeOOH Nanostructures as Efficient Visible Light Photocatalyst and Room Temperature Sensor

Ting Zhu, [1+] *Wei Li Ong,* [1+] *Liangliang Zhu* [1] *and Ghim Wei Ho* [123]*

[1] Department of Electrical and Computer Engineering, National University of Singapore, 4 Engineering Drive 3, 117583, Singapore

[2] Engineering Science Programme, National University of Singapore, 9 Engineering Drive 1, 117575, Singapore

[3] Institute of Materials Research and Engineering, A*STAR (Agency for Science, Technology and Research), 3 Research Link, 117602, Singapore

[+] Contribute equally

*Corresponding author: A/Prof. Ghim Wei Ho Email: elehgw@nus.edu.sg

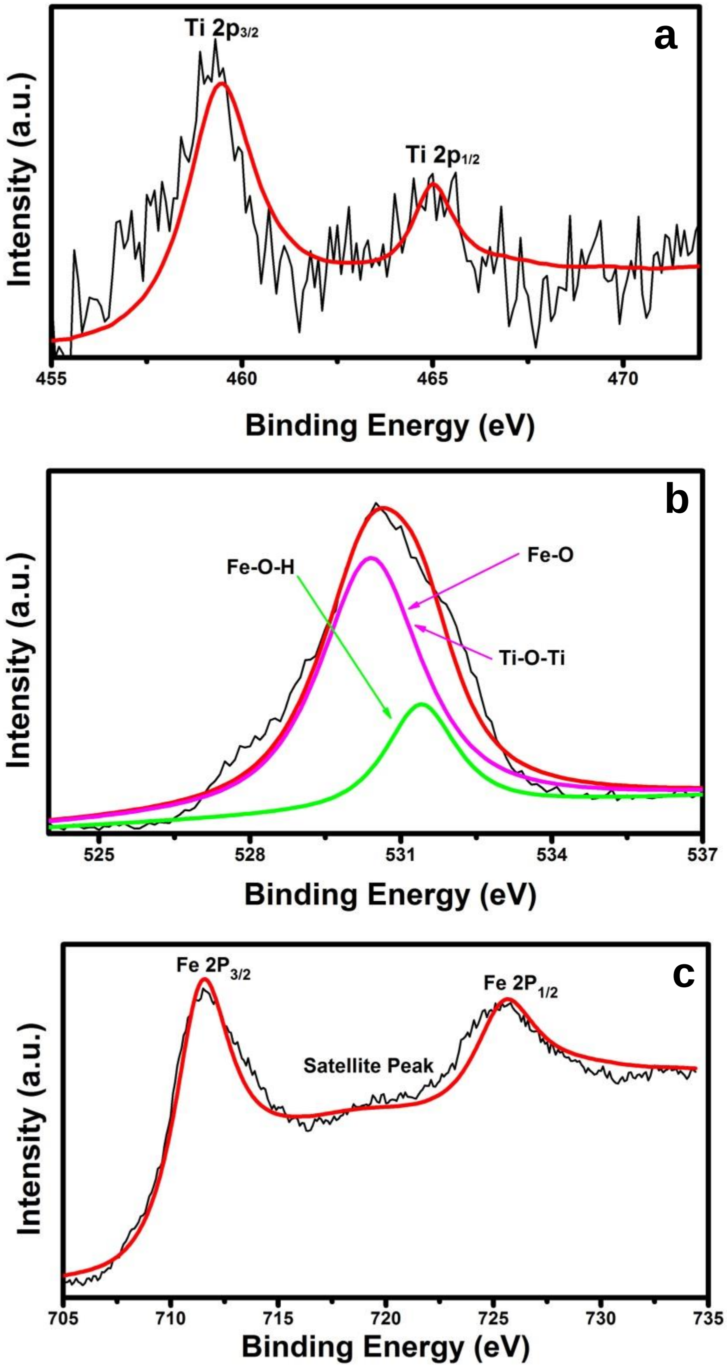

Figure S1 | XPS results of the typical TF-F sample after UV-vis photocatalysis.

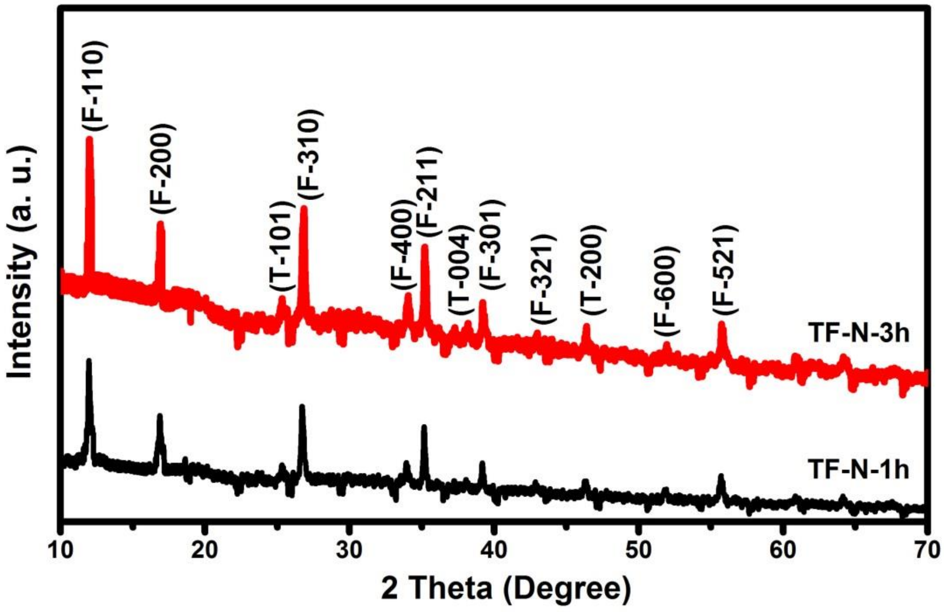

Figure S2 | XRD patterns of the TF-N samples irradiated under UV-vis after 1 and 3 h.

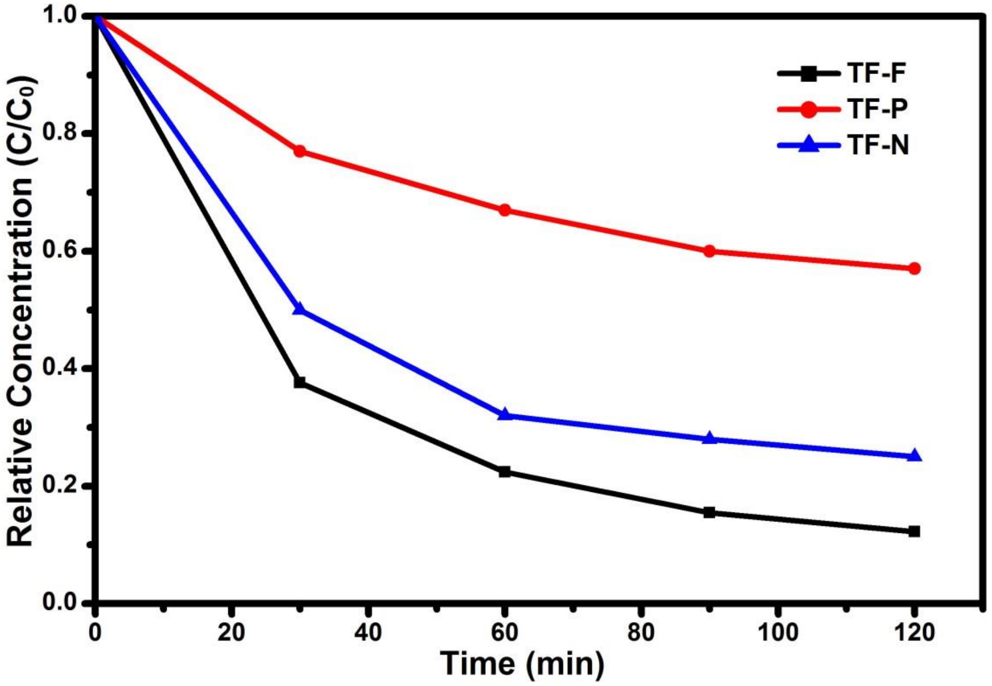

Figure S3 | Degradation kinetics of the TF samples under UV-vis irradiation without addition of H_2O_2.

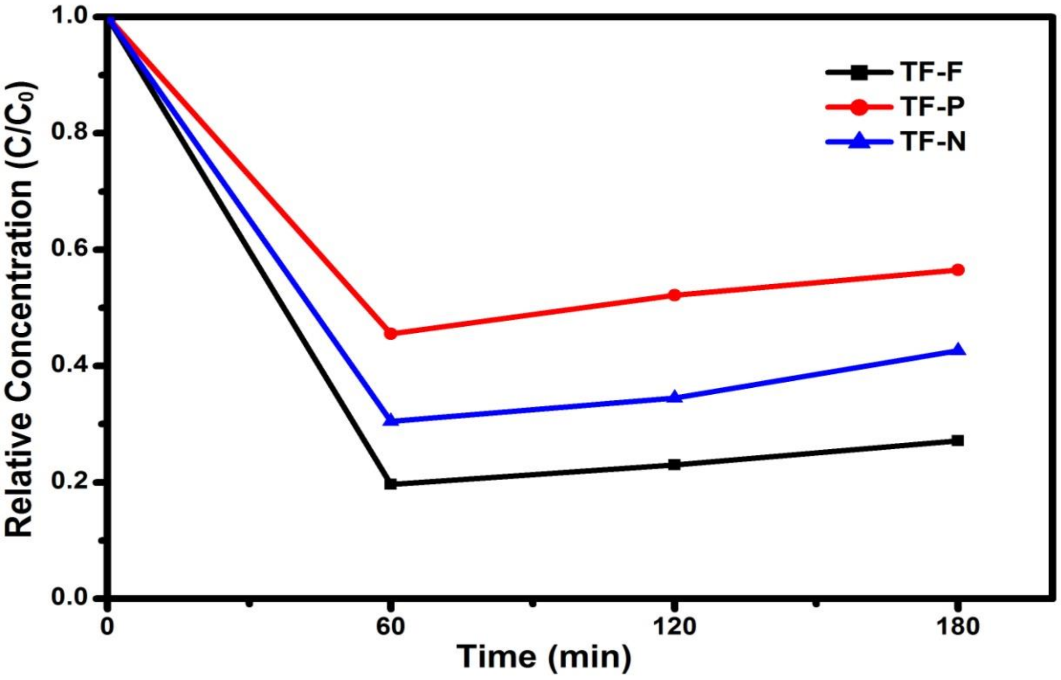

Figure S4 | Adsorption property of the TF samples without light irradiation.
